# Dengue health emergency in Peru: need for a budget allocation in line with the epidemiological scenario

**DOI:** 10.17843/rpmesp.2023.403.13117

**Published:** 2023-09-26

**Authors:** Nilthon Pisfil-Benites, Stalin Vilcarromero, Diego Azañedo

**Affiliations:** 1 Universidad Tecnológica del Perú, Chiclayo, Peru. Universidad Tecnológica del Perú Universidad Tecnológica del Perú Chiclayo Peru; 2 Edgardo Rebagliati Martins National Hospital, EsSalud, Lima, Peru. Edgardo Rebagliati Martins National Hospital EsSalud Lima Peru; 3 Universidad Científica del Sur, Lima, Peru. Universidad Científica del Sur Universidad Científica del Sur Lima Peru

To the editor. The Americas region is currently facing a health emergency due to dengue ^1^. According to the Peruvian Ministry of Health (MINSA), Peru is going through the worst health crisis on record in terms of incidence and mortality due to this disease. Reports up to the 22nd epidemiological week of the year 2023 show 201 deaths, an incidence of 130,826 cases, and presence in 20 of the 24 departments nationwide ^2^. The most affected departments are Piura, Lambayeque and Ica ^2^.

Supreme Decree 096-2023-EF was published on May 20, 2023 in response to this emergency situation, authorizing a public sector budget transfer for fiscal year 2023, in favor of MINSA, the National Health Institute (INS) and several regional governments, charged to the Contingency Reserve of the Ministry of Economy and Finance (MEF), with the purpose of funding short-term response actions at the national level ^3^. The approved transfer was S/ 34,010,329.00 for the above-mentioned institutions.

In order to evaluate the relevance of the budget allocation of SD 096-2023-EF, we carried out this analysis at the departmental level to compare the allocation of resources and the incidence and lethality of dengue. The budget allocation was obtained from the Emergency Expenditure Execution Module - Rainfall of the Ministry of Economy and Finance web portal ^4^. We used budget information regarding the activity “Evaluation, diagnosis and treatment of metaxenic diseases” at the national government level (S/ 5,136,690.00), and regional governments (S/ 10,867,420.00), together with their corresponding executing units. Likewise, departmental data on incidence per 100,000 inhabitants and dengue lethality rates were obtained from the Dengue Daily Situation Room, accessed on June 6, 2023 ^5^. In order to properly compare the resources allocated to the regional governments, we calculated the per capita budget allocation within the framework of SD 096-2023-EF, taking into account the departmental population in 2022.

**Figure 1:**
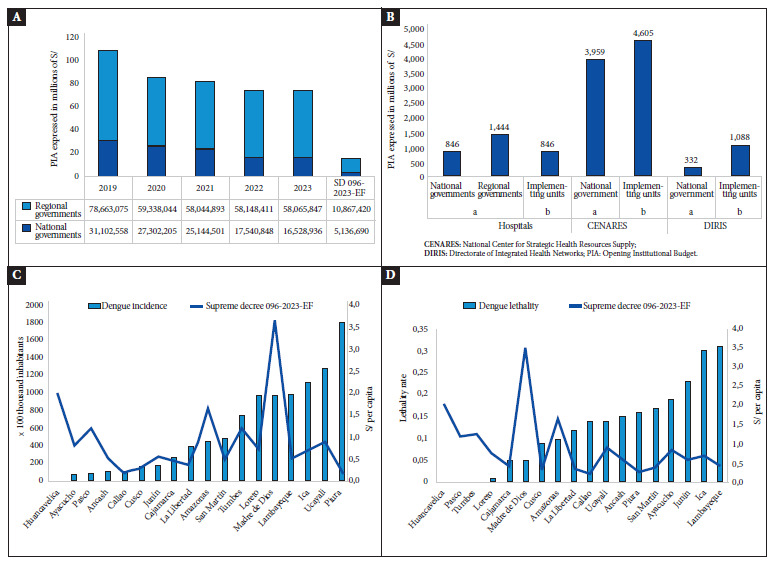
ABudget allocation of SD 096-2023-EF in the central government and some regions of Peru. (A). Comparison of the Opening Institutional Budget (PIA) during the period 2019-2023 and the budget allocation of DS 096-2023-EF, allocated for the evaluation, diagnosis and treatment of metaxenic diseases at the national government level and some regional governments of Peru. (B). Distribution of the budget allocated by SD 096-2023-EF for the product: (a) diagnosis and treatment of metaxenic diseases and (b) budget category: metaxenic diseases and zoonoses, in the different executing units, national and regional governments. (C). Distribution of the budget allocated by SD 096-2023-EF for the activity: Evaluation, diagnosis and treatment of metaxenic diseases (per capita) in regions and regional incidence (x 100 thousand inhabitants) for dengue.

We identified a decrease in the budget allocated to metaxenic diseases and zoonoses between 2019 and 2023, both in the national and regional governments (Figure 1). Most of the budget allocated to the diagnosis and treatment of metaxenic diseases was distributed among several regional governments. Most of the budget allocated according to SD 096-2023-EF was destined to the National Center for Strategic Health Resources Supply (CENARES) at both the national government level and its executing units (Figure 1).

The highest incidence rates of dengue × 100,000 population were found in Piura, Ucayali, Ica and Lambayeque, although the per capita budget allocations of these departments were among the lowest at the national level (Figure 1). The department with the highest budget allocation at the national level was Madre de Dios, which ranks fifth in terms of incidence and fourteenth in terms of fatality rate (Figure 1). The four regions with the highest dengue lethality rates (Lambayeque, Ica, Junin and Ayacucho), have the lowest per capita budgets under SD 096-2023-EF.

Our results show that the budget allocation for the “Evaluation, diagnosis and treatment of metaxenic diseases” under SD 096-2023-EF is not consistent with the population needs in terms of incidence and lethality rates in most Peruvian departments. This budget allocation problem could be due to the fact that some of the departments with the highest indicators had not had such an impact in previous years, with the exception of Piura, Lambayeque and Ica, which experienced an increasing number of cases in recent years, with the highest figures reported in 2022, as well as in 2017, when the largest nationwide epidemic associated with the El Niño Costero occurred ^2^. Therefore, the impact of dengue in these departments during 2023 was predictable, due to the climatic phenomena that occurred in the first half of the year.

Urgent measures need to be applied in order to mitigate this problem. To begin with, budget allocations for transfers or allotments should be distributed considering real-time epidemiological criteria based on adequate monitoring and evaluation of the current and changing needs of each department, such as the current distribution of cases and lethality rate. Other factors that may play a role in the high lethality rate reported this year should also be taken into account, such as the fact that dengue has spread to non-endemic areas, which must adapt and prepare their infrastructure, logistics and health personnel to face the dengue epidemic. In the meantime, specialists have been trained and deployed, along with trained health personnel, to the most affected areas of the country in order to manage the cases. All this implies a larger budget for human resources, travel expenses, laboratory and radio-diagnostic tests, as well as logistics for the implementation of hydration and hospitalization centers. Besides, in order to contain a dengue outbreak or epidemic, vector control is crucial and requires trained personnel, the use of appropriate spraying and insecticide equipment and, above all, a very large budget. Finally, massive education strategies focused on prevention should be included in this intervention plan for the most affected departments. 

In conclusion, there is a need to reallocate the budget in order to control the dengue epidemic and reduce its impact. Furthermore, future budget allocations should consider, in addition to the history of cases and regions, and real-time epidemiological and entomological surveillance, projections based on climatic phenomena such as El Niño.
