# Supplementary material for: Dengue health emergency in Peru: need for a budget allocation in line with the epidemiological scenario
Source: Rev Peru Med Exp Salud Publica. 2023 Sep 26;40(3):374–6. doi: 10.17843/rpmesp.2023.403.13117 (PMC10953675; doi:10.17843/rpmesp.2023.403.13117)
Supplement: Translation

# Translation: Emergencia sanitaria por dengue en el Perú: necesidad de una asignación presupuestal acorde a la situación epidemiológica

**Authors:** Nilthon Pisfil-Benites, Stalin Vilcarromero, Diego Azañedo

**Affiliations:** 1 Universidad Tecnológica del Perú, Chiclayo, Perú.; 2 Hospital Nacional Edgardo Rebagliati Martins, ESSALUD, Lima, Perú.; 3 Universidad Científica del Sur, Lima, Perú.

Sr. Editor. Actualmente, la región de América enfrenta una emergencia sanitaria debido al dengue ^1^. Según el Ministerio de Salud (MINSA) de Perú, estamos atravesando la peor crisis sanitaria registrada en términos de incidencia y mortalidad por esta enfermedad. Hasta la semana epidemiológica 22 del año 2023, se han registrado 201 defunciones, una incidencia de 130 826 casos, y un alcance en 20 de los 24 departamentos a nivel nacional ^2^. Asimismo, los departamentos más afectados son Piura, Lambayeque e Ica ^2^.

Como respuesta a esta emergencia, el 20 de mayo del presente año se publicó el Decreto Supremo 096-2023-EF, que autoriza una transferencia presupuestal del sector público para el año fiscal 2023, a favor del MINSA, el Instituto Nacional de Salud (INS) y los diversos gobiernos regionales, con cargo a la Reserva de Contingencia del Ministerio de Economía y Finanzas (MEF), con el propósito de financiar las acciones de respuesta a corto plazo a nivel nacional ^3^. La transferencia aprobada fue de S/ 34,010,329.00 para las instituciones mencionadas.

Con el objetivo de evaluar la pertinencia en la asignación presupuestal del DS 096-2023-EF, se realizó el presente análisis a nivel departamental comparar la asignación de recursos con las tasas de incidencia y letalidad del dengue.. La asignación presupuestal se obtuvo del módulo de ejecución del gasto de emergencias - Lluvias del portal web del Ministerio de Economía y Finanzas ^4^. Se utilizó la información presupuestal de la actividad «Evaluación, diagnóstico y tratamiento de enfermedades metaxénicas» a nivel del gobierno nacional (S/ 5,136,690.00), y gobiernos regionales (S/ 10,867,420.00), conjuntamente con sus respectivas unidades ejecutoras. Asimismo, se utilizaron los datos departamentales de incidencia por 100,000 habitantes y las tasas de letalidad por dengue, obtenidos de la Sala situacional diaria de Dengue, consultada el 06 de junio de 2023 ^5^. Para realizar una comparación adecuada de los recursos asignados entre los gobiernos regionales, se calculó la asignación presupuestal per cápita en el marco del DS 096-2023-EF, tomando en cuenta la población departamental del 2022.

Se identificó una disminución del presupuesto asignado a las enfermedades metaxénicas y zoonosis entre los años 2019 y 2023, tanto en el gobierno nacional como regionales (Figura 1). En cuanto a la asignación de presupuesto para el diagnóstico y tratamiento de enfermedades metaxénicas, la mayoría de los recursos fueron distribuidos en diversos gobiernos regionales. La mayor parte del presupuesto asignado según el DS 096-2023-EF fue destinado al Centro Nacional de Abastecimiento de Recursos Estratégicos en Salud (CENARES) tanto a nivel del gobierno nacional como sus unidades ejecutoras (Figura 1).

**Figura 1:** Asignación presupuestal del DS 096-2023-EF en el gobierno central y algunas regiones del Perú. (A). Comparación del Presupuesto Institucional de Apertura (PIA) durante el período 2019-2023 y la asignación presupuestal del DS 096-2023-EF, asignados para la evaluación, diagnóstico y tratamiento de enfermedades metaxénicas a nivel de gobierno nacional y algunos gobiernos regionales del Perú. (B). Distribución del presupuesto asignado por DS 096-2023-EF para el producto: (a) diagnóstico y tratamiento de enfermedades metaxénicas y (b) la categoría presupuestal: enfermedades metaxénicas y zoonosis, en las diferentes unidades ejecutoras, gobierno nacional y regionales. (C). Distribución del presupuesto asignado por DS 096-2023-EF para la actividad: Evaluación, diagnóstico y tratamiento de enfermedades metaxénicas (per cápita) en regiones e incidencia (x 100 mil habitantes) regional por dengue. (D). Distribución del presupuesto asignado por DS 096-2023-EF para la actividad: Evaluación, diagnóstico y tratamiento de enfermedades metaxénicas (per cápita) y porcentaje de letalidad regional por dengue.

A nivel departamental las mayores incidencias de dengue × 100 000 habitantes se identificaron en Piura, Ucayali, Ica y Lambayeque, aunque las asignaciones presupuestales per cápita en estos departamentos, están entre las más bajas a nivel nacional (Figura 1). El departamento con la asignación presupuestal más alta a nivel nacional fue Madre de Dios, que ocupa el quinto lugar en términos de incidencia y el decimocuarto lugar en términos de letalidad (Figura 1). Las cuatro regiones con mayor letalidad por dengue (Lambayeque, Ica, Junín y Ayacucho), tienen los presupuestos per cápita más bajos en el marco del DS 096-2023-EF.

Nuestros resultados muestran que la asignación presupuestal para la «Evaluación, diagnóstico y tratamiento de enfermedades metaxénicas» en el marco del DS 096-2023-EF, no es coherente con las necesidades en términos de incidencia y letalidad en la mayoría de los departamentos peruanos. Este problema de asignación presupuestal podría deberse a que algunos de los departamentos que ahora muestran las cifras más altas de indicadores, no habían tenido tal impacto en los años anteriores, con excepción de Piura, Lambayeque e Ica que presentaron incremento de casos en los últimos años, con las cifras más altas en el 2022, así como en el año 2017, cuando ocurrió la epidemia más grande a nivel nacional asociado al Fenómeno de El Niño Costero ^2^. Por lo tanto, el impacto del dengue en esos departamentos durante el 2023 era previsible, debido a los fenómenos climáticos ocurridos en la primera mitad del año.

Existe la necesidad de tomar medidas urgentes para mitigar este problema. En primer lugar, la asignación presupuestal por transferencias o habilitaciones, deben ser distribuidas considerando criterios epidemiológicos en tiempo real que deben estar basados en un adecuado monitoreo y evaluación de las actuales y cambiantes necesidades de cada departamento, tales como la actual distribución de casos y la tasa de letalidad. Asimismo, se debe tomar en cuenta otros factores que pueden jugar un rol en la letalidad alcanzada en el presente año, como el hecho de que la enfermedad del dengue se ha extendido a zonas no endémicas cuya infraestructura, logística y personal de salud deben adaptarse y prepararse para afrontar esta epidemia de dengue. Mientras tanto, han sido necesarios la capacitación y el despliegue de especialistas y de más personal de salud a las zonas más afectadas del país para el manejo adecuado de los casos. Todo esto implica mayor presupuesto en recursos humanos, viáticos, exámenes de laboratorio y radiodiagnóstico, logística para la implementación de centros de hidratación u hospitalización. Pero, además, se sabe que, para la contención de un brote o epidemia de dengue, el control vectorial es determinante y requiere de personal capacitado, uso de equipo de fumigación e insecticida adecuados. Por último, dentro de este plan de intervención en los departamentos más afectados, debe incluirse la educación masiva para prevenir el dengue.

En conclusión, existe la necesidad de realizar una reasignación del presupuesto para el control de la epidemia del dengue y disminución de su impacto. Asimismo, las asignaciones presupuestales futuras deben considerar además del histórico de casos y regiones, y la vigilancia epidemiológica y entomológica en tiempo real, las proyecciones basadas en fenómenos climáticos como El Niño.
